# Patient perceptions of nurses’ cultural competence in public sector hospitals in Gauteng

**DOI:** 10.4102/hsag.v29i0.2499

**Published:** 2024-06-14

**Authors:** Disebo R. Maboko, Sue Armstrong, Daleen Casteleijn

**Affiliations:** 1 Department of Nursing Education, Faculty of Health Sciences, University of the Witwatersrand, Johannesburg, South Africa; 2 Department of Occupational Therapy, Faculty of Health Sciences, University of the Witwatersrand, Johannesburg, South Africa

**Keywords:** cultural competence, patient perceptions, public health sector, nurses, Gauteng

## Abstract

**Background:**

Healthcare institutions are increasingly receiving patients from diverse cultural backgrounds because of migration, rapid urbanisation, and easier access to healthcare. Because the satisfaction of these patients is linked to their perceptions of appropriate cultural care, understanding patient perspectives about cultural competence is imperative. Additionally, patient perceptions about nurses’ cultural competence are largely unexplored in South Africa.

**Aim:**

This study explored how the concept of cultural competence is perceived by patients.

**Setting:**

Three public sector hospitals in Gauteng, one from each of the three different levels of public sector hospitals – district (level one), regional (level two), and academic (tertiary, level three).

**Methods:**

This study derives from the qualitative phase of a larger sequential exploratory mixed methods study. The study population was patients in public sector hospitals. A total of 21 interviews were conducted after purposive stratified sampling was done. Data analysis followed Tesch’s eight steps of data analysis.

**Results:**

Patients in public sector hospitals in Gauteng believe consideration of culture is important in nursing. They identified the cultural needs they would like nurses to acknowledge, such as being asked about their food preferences and mentioned the need to evaluate nurses’ level of cultural competence.

**Conclusion:**

Patient perceptions about cultural competence and their cultural needs can assist nurses in gauging how culturally competent they are and improving care to patients.

**Contribution:**

Patients’ perceptions revealed that nurses must be competent to acknowledge their specific cultural needs such as food, language preferences, and religious practices.

## Introduction

Upon graduation, South African nurses, as part of the pledge of service, make a solemn promise not to discriminate against patients based on religion, nationality, race, or social standing (South African Nursing Council 2004–2019). Therefore, these nurses should not discriminate against patients who are different from them but rather render culturally sensitive nursing care and cater to their diverse needs. According to the South African Bill of Rights, no person is permitted to discriminate against another based on race, gender, sex, pregnancy, marital status, ethnic or social origin, colour, sexual orientation, age, disability, religion, conscience, belief, culture, language, and birth (*South African Government, Act No. 108 of 1996*). While these legal and ethical guidelines help to direct the conduct of nurses when providing care to patients, they do not make diversity management in nursing units any less complex.

Dwamena et al. (2012) state that communication problems in healthcare can occur when health professionals choose to focus on diseases and their management rather than on people, their lives, their background, and their contexts. One important concept to focus on during healthcare delivery is the issue of cultural competence. These authors suggest that the more culturally competent nurses are, the more patients will be satisfied with the care they receive. Campinha-Bacote (2002) states that there is a global challenge for healthcare providers to prioritise cultural competence because of changing demographics and economics, and the longstanding inequalities in health status in our world. Thus, cultural competence should be an important focus for nurses, and in fact, all healthcare professionals in a diverse nation like South Africa.

South Africa is a culturally diverse nation and what compounds the complexities are the socio-political landscape, growing inequality and poverty in neglected communities, increasing urbanisation, and growth in migrants, especially from other African countries. This situation has resulted in about 35 million (63.6%) people immigrating to urban areas, like the province of Gauteng where this study took place. At a social level, South Africa faces several service delivery challenges because of shortages of health professionals (Matthews & Van Wyk 2018). If the staff establishment of a healthcare facility does not resemble the community that it serves, patients will likely be dissatisfied with the care they receive on account of language barriers, and they will feel misunderstood by the staff members. According to Matthews and Van Wyk (2018), many healthcare services still have pronounced racial and ethnic inequalities dating back to the apartheid era. In addition, research on cultural competence and educating health professionals about culturally competent care is limited.

Furthermore, there is a great need for research in the field of cultural competence linked to healthcare in South Africa. Internationally, there is a large body of literature devoted to cross-cultural challenges faced by nurses and the cultural competence of nurses and student nurses. However, most of the studies conducted on nurses focus more on their own perceived cultural competence and less on patients’ perceptions about their cultural competence or on understanding patients’ cultural needs (Almutairi, McCarthy & Gardener 2015; Jeffreys 2016; Repo et al. 2017). Understanding patient perceptions about cultural competence could assist nurses in becoming more culturally sensitive during patient care. This will also provide a yardstick against which the nurses can measure their cultural competence.

Most studies on cultural competence in the nursing profession have been conducted outside the South African context. According to Tavallali, Kabir and Jirwe (2014), the concept of cultural competence has been studied by numerous international theorists and countries including Sweden (Jirwe 2008), the United States of America (US) (Campinha-Bacote 2002), England (ed. Papadopoulos 2006), and New Zealand (Papps 2005). The Swedish researchers identified the most important components of cultural competence as awareness of diversity among human beings and a non-judgemental openness towards all individuals according to Tavallali et al. (2014). The authors further discuss another Swedish study, which considered the cultural competence of nurses and students and indicated that this includes cultural understanding, cultural sensitivity, and cultural encounters. Jirwe et al. (2009) in their study, aimed at identifying the core components of cultural competence, identified components that were categorised into the following five areas: cultural sensitivity, cultural understanding, cultural encounters, understanding of health, ill health, and healthcare and social and cultural contexts. These authors concluded that there were some similarities between what is raised in their study and existing frameworks of cultural competence from the US and the United Kingdom (UK). However, Swedish experts placed less emphasis on ethno-history and on developing skills to challenge discrimination and racism.

South Africa has historically not been a good example of non-judgemental openness towards all individuals even though so much diversity exists in this nation. There have certainly been efforts to challenge discrimination and racism in South Africa; however, more still needs to be done in this regard. It is important to establish how the concept of cultural competence is understood in the South African context, including exploring patients’ perceptions about the issue. Although there is consensus among scholars that cultural competence in nurses improves patient outcomes, very few studies have focussed on patient perceptions and patient outcomes related to cultural competence (Alizadeh & Chavan 2015; Tavallali et al. 2014).

Several conceptual models and frameworks have been developed in both the healthcare and business sectors to describe the components of cultural competence (Alizadeh & Chavan 2015). The authors point out that there are several well-developed business models and frameworks that can be utilised in healthcare; however, it is also important for healthcare professionals to use discipline-specific models that suit the context of their studies as there are some differences in definitions and components of cultural competence between these models. In nursing, the concept of cultural competence originates from Leininger’s Theory of Culture Care Diversity and Universality. This theory was developed from Leininger’s work on transcultural nursing in 1991. Various models translate this theory into cultural competence in nursing practice (Garneau & Pepin 2015; McFarland & Wehbe-Alamah 2019).

Different models were developed in nursing to translate Leininger’s theory into practice and present factors that could influence cultural competence, and of these, the definition of cultural competence most commonly cited in the literature is the one from Campinha-Bacote’s model developed in 1999 (Garneau & Pepin 2015), known as The Process of Cultural Competence in the Delivery of Healthcare Services, developed in the US (Campinha-Bacote 2002). According to Campinha-Bacote (2002:181), cultural competence is defined as the ongoing process in which the healthcare provider continuously strives to achieve the ability to effectively work within the context of the client, individual, family or community.

**FIGURE 1:**
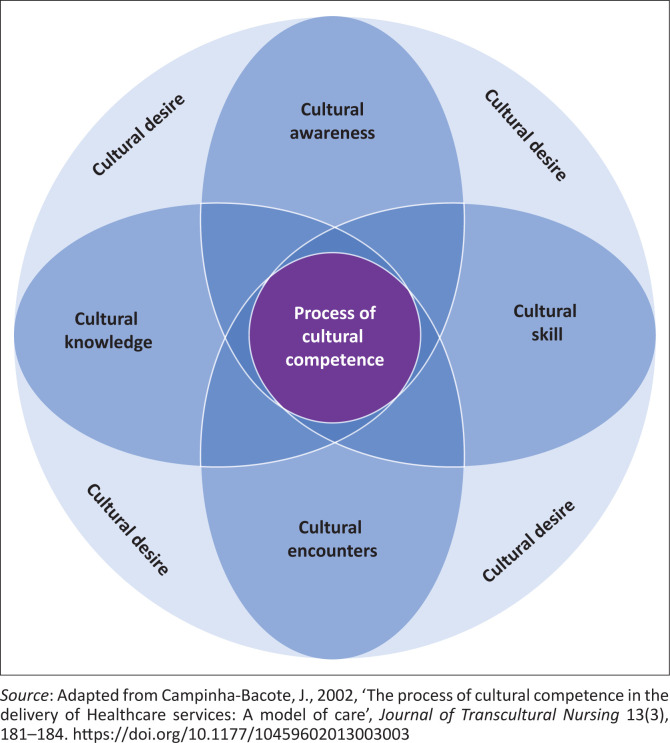
The process of cultural competence in the delivery of health care services.

This study was underpinned by the Process of Cultural Competence in the Delivery of Healthcare Services model, as this model assumes that there is a direct relationship between the level of cultural competence of healthcare practitioners and their ability to provide culturally responsive services. Thus, cultural competence is seen as a process that should be responsive across variation both within and across groups of culturally and ethnically diverse clients (Campinha-Bacote 2002). The model presented by Campinha-Bacote (2002:18) consists of five constructs: cultural awareness, cultural knowledge, cultural skill, cultural encounters, and cultural desire. These constructs have an interdependent relationship with each other, and it is the intersection of these constructs that depicts the true process of cultural competence in healthcare (Figure 1). In meeting the aim of the study, the model was used as a theoretical framework to guide this study. The model was used to formulate the questions in the semi-structured interview guide used during data collection and as a template during data analysis. The discussion of the results of the study also reflected on the model.

Table 1 defines the constructs of the model. The reader is referred to these definitions as they are used in the findings and discussion of this article.

**TABLE 1:** Definitions of the cultural constructs as developed.

Cultural constructs	Definition
Cultural awareness	The in-depth self-assessment and reflection on an individual’s cultural and professional background to avoid the risk of imposing one’s culture on others.
Cultural knowledge	The process in which a sound knowledge of other people’s cultures is acquired by healthcare providers.
Cultural skill	The process of collecting relevant cultural data regarding a client’s presenting problem and performing a culturally based physical assessment.
Cultural encounters	The opportunities health care providers expose themselves to, to acquire knowledge about different cultures.
Cultural desire	The desire by a healthcare provider to be culturally competent without being compelled to do so.

*Source*: Adapted from Campinha-Bacote, J., 2002, ‘The process of cultural competence in the delivery of Healthcare services: A model of care’, *Journal of Transcultural Nursing* 13(3), 181–184. https://doi.org/10.1177/10459602013003003

Nurses are bound ethically and legally to care for patients irrespective of religion, nationality, race, or social standing. It is therefore imperative that nurses ensure that patients receive culturally sensitive care. It is challenging for nurses to care for patients from diverse backgrounds, and if these challenges are not addressed, they could contribute to poor-quality patient care. According to the researcher’s experience, diversity management, including cultural diversity management tends to be neglected in organisations, including healthcare facilities because of the difficulties associated with addressing these issues. Many healthcare services still have pronounced racial and ethnic inequalities dating back to the apartheid era (Matthews & Van Wyk 2018). The issues of race and diversity management are a problem in other countries as well. Cronin (2014) states that in the UK, foreign patients or patients from marginalised groups face challenges related to diversity and inequality. The issues faced by foreigners and refugees in the UK are not new as a study by Papadopoulos et al. (2004) showed that some Ethiopian refugees found it difficult to access health services because of language problems and poor understanding of the primary healthcare system. Because the perceptions of patients about the cultural competence of nurses have not been adequately explored, this study aimed to explore and describe how the concept of cultural competence of nurses is perceived by patients in some public sector hospitals in Gauteng.

## Research methods

### Study setting

The study was conducted in three public hospitals in the Gauteng province all in the Johannesburg region. Participants from one district hospital (level one hospital offering minimal healthcare services), one regional hospital (level two hospital that offers slightly more advanced healthcare services like the intensive care unit), and one academic hospital (tertiary, level three or major hospital offering the highest level of care and widest range of services) in this region were included in the study.

### Study population and sampling

The study population comprised of patients in public healthcare facilities in Gauteng. The sampling method used was purposive and stratified using a sampling frame to include participants who were knowledgeable about the subject and ensured different types of hospitals and cultures respectively, were included. A minimum of seven participants were included in each of the three hospitals in which the study was conducted. The sampling frame assisted the researcher in approaching patients of different races, ethnic groups, religions, and home languages. Patients were requested to self-identify their cultures. A sociologist was consulted to ensure that the sampling was adequately stratified to ensure that the demographics of the sample were similar to those of the population of the Gauteng province. All selected participants who met the inclusion criteria were approached personally to ask for their permission to participate in the pharmacies or wards of the participating hospitals. Adult patients (18 years and older and able to give consent) who had been admitted at least once and discharged or recovering inpatients from different cultural groups who were willing and physically able to participate from the participating hospitals, were included. Most of the participants were outpatients because they were more accessible when compared to inpatients as some inpatients were unable to participate because of still being ill (bedbound or unable to mobilise to the interview room).

### Data collection

**FIGURE 2:**
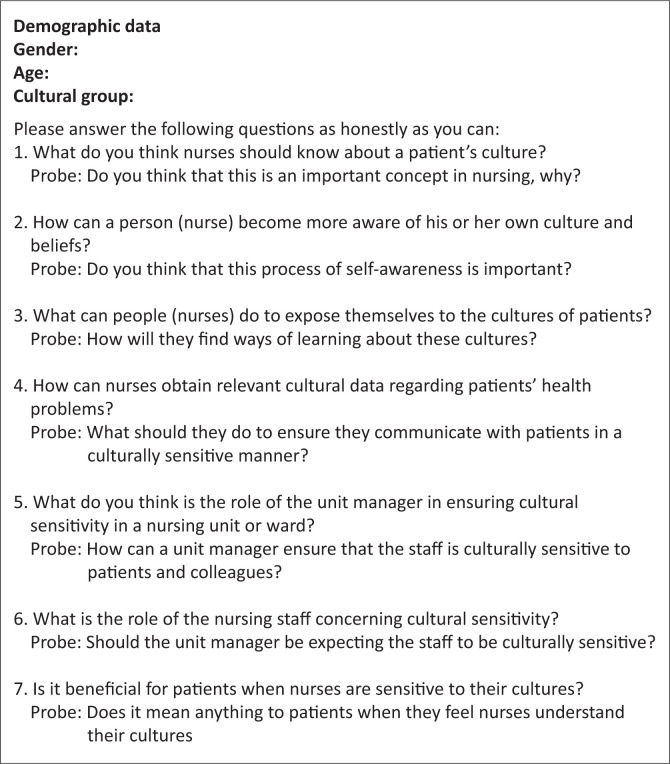
Semi-structured interview guide for patient interviews.

The researcher conducted this study as part of her PhD study and had no relationship with any of the study participants. Data was collected by the researcher herself, who had an MSc in Nursing degree and previous experience with conducting qualitative interviews. Face-to-face interviews were conducted using a semi-structured interview guide (Figure 2) with questions based on the theoretical framework and the aim of the study.

The first interview was used as a pilot test; however, its results were included in the main study as its results showed that nothing needed to change in the semi-structured interview guide or the process of data collection. The interviews were conducted in English; however, some participants who struggled to express themselves in English were allowed to use Zulu for some parts of the interview. Interview techniques such as listening, paraphrasing, among others, were used during the interviews. The interviews were audio-recorded and conducted after informed written consent was obtained from the participants. To obtain informed written consent from the study participants, they were given an information letter and consent forms. These documents contained information on the study and how their rights would be upheld. The interviews were conducted in a private and quiet room, without any disturbance, lasting no more than an hour. During the interviews, handwritten field notes were also made in a notebook. According to Gray, Grove and Sutherland (2017), the sample size in qualitative research is determined by data saturation, a point reached when participants start to repeat the same information. Data were collected until data saturation was achieved after 21 interviews.

### Data analysis

Interviews were transcribed (and some translated) verbatim by a transcription company and the researcher went through all transcripts while listening to the recordings to check for any transcription and translation errors. Data analysis was done concurrently with data collection as the interview data were sent to the transcription company immediately following the first seven interviews and the rest were sent as soon as the first seven were received back. Analysis of each transcript was done as soon as it was received back from the transcription company which took an average of 4 days each. Some delays were encountered; therefore, listening to the interview recordings was done after each interview while waiting for the transcripts to arrive back to make it easier to analyse the transcripts when they arrived back. The transcripts were analysed according to Tesch’s eight steps of data analysis as explained in (Creswell & Creswell 2018), manually without the use of any software. These steps were used as follows:

Firstly, all the transcripts were read thoroughly and carefully and ideas that came to mind were written down. Secondly, one transcript was selected at a time, and questions such as ‘What is this about?’ and ‘What is important or of value in the information gathered?’ were asked while going through the transcript and writing down thoughts in the margin of every transcript. Thirdly, a list of topics was compiled from the data and abbreviated as codes, written next to the appropriate parts of the text, in step four. In step five, the most descriptive words for topics were used and these were sorted into categories. The number of categories was reduced by grouping related topics and drawing lines between the categories to show how they were interrelated. In step six, themes were developed from these categories followed by assembling data parts that belonged to each theme in one place, performing an initial analysis of the themes in step seven and lastly, recoding the data was done to finalise the themes (Tesch 1990; Creswell & Creswell 2018:310). The study supervisors were also involved in co-coding and finalising the themes of the study. Field notes were read during the data analysis process to keep in mind the context, thoughts, and facts jotted down during data collection.

### Measures of trustworthiness

Trustworthiness in this study was ensured by implementing credibility, transferability, dependability, and confirmability as defined by Babbie and Mouton (2001) and Shenton (2004). To ensure credibility, referential adequacy was implemented by audio-recording the interviews and transcribing the data verbatim. Transferability and dependability were ensured by describing the data collection methods in detail. Dependability was also ensured through the research supervisors guiding the data collection and analysis processes. To ensure confirmability, an audit trail (an honest description of the steps taken from the start of a research study to the reporting of findings) was kept through raw data, field, and data analysis notes. These notes also allowed for reflexivity to occur (Babbie & Mouton 2001; Shenton 2004).

### Ethical considerations

Ethical clearance to conduct this study was obtained from the University of the Witwatersrand, Human Research Ethics Committee (No.M140633).

Permission to conduct the study was also obtained from all three hospitals in which the study was conducted. Informed written consent was obtained from all the participants in this study. Informed written consent for audio-recording the interviews was also obtained. The methods of the study were clearly explained, and adhered to, to avoid any deception in the processes followed and how they were reported. To respect the participants’ right to self-determination or autonomy, the participants were not coerced or deceived into participating in the study. Instead, they were given all the information they needed to make an informed decision. Informed consent was sought from all the study participants by offering them an information letter. The data was only accessible to the researcher, study supervisors, and the transcription company used to transcribe and translate the interviews, as it was kept in a locked cupboard to uphold the participants’ right to privacy. The transcribers of the transcription company were required to sign confidentiality agreements with the transcription company. The identity of the participants was not revealed to anyone; instead a reference number was used to refer to the participants to maintain their anonymity.

## Results

The 21 patients who participated in this study represented the different races living in South Africa as follows: 52.38% were African, 9.5% Caucasian, mixed-race, and 19.05% were Indian participants. Their ages ranged between 22 and 81 years. Regarding gender, 28.6% were male and 71.4% female. Most of the participants (81%) were outpatients who were recruited at the pharmacy of the hospital where they were collecting their monthly chronic condition medication. The rest of the participants amounting to 19% were recruited from surgical wards of one hospital (hospital B) in which they were inpatients.

In terms of religion, 85.7% were Christian, 9.5% Muslim, and 4.8% Hindu. The nationality of the participants was 85.7% South African and 14.3% originating from neighbouring African countries. All the participants could speak English, with 4.8% being unilingual, whereas 95.2% were bi- or multi-lingual – because of also speaking either one South African or african language or more languages (for the african participants) or Afrikaans (for the mixed race and caucasian participants). All the participants in this study had the experience of being admitted to a hospital during their chronic illnesses (Table 2).

**TABLE 2:** Patient demographics.

Participant code	Gender	Age (years)	Self-identified culture	Race	Religion	Main language or languages	Type of patient
P1	Female	52	South African or Mixed race	Mixed-race person	Christian	English and Afrikaans	Outpatient
P2	Female	71	European	Caucasian person	Christian	English	Outpatient
P3	Female	60	Hindu (Tamil)	Indian person	Hindu	Tamil and English	Outpatient
P4	Female	37	Tswana	African person	Christian	Tswana	Outpatient
P5	Male	81	Zulu clan name	African person	Christian	Zulu	Outpatient
P6	Male	48	Mixed race	Mixed-race person	Christian	English and Afrikaans	Outpatient
P7	Male	53	Muslim	Indian person	Muslim	English mostly	Outpatient
P8	Female	47	Zulu	African person	Christian	Zulu	Outpatient
P9	Female	62	Afrikaans and English or South African	Caucasian person	Christian	Afrikaans and English	Outpatient
P10	Male	31	Lomwe	African person	Christian	Lomwe	Outpatient
P11	Female	30	Shona	African person	Christian	Shona	Inpatient
P12	Female	60 or 63	Christian	Indian person	Christian	English	Inpatient
P13	Male	26	Christian	African person	Christian	Kalanga	Inpatient
P14	Male	22	Swati	African person	Christian	Swati	Inpatient
P15	Female	41 or 42	Christian	African person	Christian	Sepedi	Outpatient
P16	Female	35	Combination of black and white culture	African person	Christian	Zulu and English	Outpatient
P17	Female	57	Christian	African person	Christian	Southern Sotho	Outpatient
P18	Female	51	Venda	African person	Christian	Venda	Outpatient
P19	Female	48 or 58	Muslim	Mixed-race person	Muslim	English and Afrikaans	Outpatient
P20	Female	72	Mixed race	Mixed-race person	Christian	English and Afrikaans	Outpatient
P21	Female	59	Christian	Indian person	Christian	English	Outpatient

The demographics of the participants in this study were consistent with the demographics of the population of the Gauteng province and City of Johannesburg as reported by Statistics South Africa (2011).

Table 3 shows the themes, subthemes (categories) concepts, and verbatim quotes from the participants.

**TABLE 3:** Themes, subthemes and concepts.

Themes	Subthemes or categories	Concepts and verbatim quotes
Patients’ understanding of culture	Description of culture and its importance	**Identification of culture** ‘I would usually say South African, ne [*right*] … Ya [*yes*], a so-called coloured [*mixed race*] Yes, it is very, very important.’ (P1, Mixed race, Christian)
	Values or norms	**Food, Dress code, Respect, Kindness** ‘Muslims, they don’t eat certain food and if you know that I don’t eat pork, I don’t eat anything that’s cooked out of a Christian’s pot, that’s my culture …’ (P7, Mixed race, Muslim)
Importance of cultural competence in nursing	Nurses’ cultural knowledge	**Knowledge about patients’ cultures** ‘Yes it is very important … they will find out about how the Muslim culture lives [*the Muslim patients’ lifestyle*].’ (P7, Indian, Muslim)
	Nurses’ self-awareness	**Nurses to understand their own cultures** ‘So they know themselves better and that will help them in understanding other people’s cultures.’ (P9, Caucasian, Christian)
	Cultural needs do change	**Re-definition of culture** ‘We are not to be disrespectful in any way but with modern times things has [*have*] changed. Our dressings are different. Our talking is different because we used to talk the mother language but nobody talks the mother language anymore.’ (P3, Indian, Hindu)
Meeting patients’ cultural needs	Cultural skill	**Asking patients about important cultural information** ‘Like what type of food do you eat? … because different cultures eat different food.’ (P14, African, Christian)
	Cultural encounters	**Making time to talk to patients** ‘It’s to communicate to the patient or person, communication is playing [*plays*] a good role because then we will know each other understanding each other.’ (P1, Mixed race, Christian)
	Cultural practices or beliefs	**Giving patients privacy for cultural practices** ‘[*I*] need, the nurse must respect my culture and the things that I want to do, maybe when I come to the hospital and I know that maybe I’m going to stay for two days and I must carry my bible in the morning or just like a … in the night I can read my bible and maybe I must be free of the people of church when they visit me, I must be comfortable.’ (P20, Mixed race, Christian)
	Role of the nurses and unit managers	**Managers need to supervise nurses and nurses should be culturally competent** ‘So the role of the sister in charge in the ward, she might see if the nurses are treating the patient okay [*well*], alright, and then she must also take care of [*it*] if the patient complains, she must do her best to help.’ (P15, African, Christian)
	Benefits for the patients and nurses	**Patient satisfaction, Improved knowledge about patients’ cultures** ‘[*W*]hat I know of, it will make things even better, not only just for the patient but obviously the nurse will get to know more about the patient as well. That’s how the nurse starts learning different cultures in fact.’ (P7, Indian, Muslim)
Evaluation of nurses’ cultural competence	Some satisfaction for Christians	**Some needs of Christians are met** ‘When they see me read my bible, they don’t have a problem with that.’ (P11, African, Christian)
	Dissatisfaction with nurses’ cultural competence	**Unmet cultural needs or discrimination** ‘[*B*]ecause he’s light of [*in*] complexion, they didn’t even had [*have*] a …, they treated him so bad, I had such a bad experience with that [*those*] nurses.’ (P1, Mixed race, Christian)

The following four themes emerged from the data collected in the study: meaning of culture, importance of culture in nursing, patients’ cultural needs, and evaluation of nurses’ cultural competence. There were also subthemes and concepts derived under each theme, with supporting verbatim quotes from the participants. The subthemes were grouped according to their central focus to form a theme. The themes are the overall descriptions of the combined subthemes (Table 3).

### Theme 1: Patients’ understanding of culture

Patients in this study emphasised the importance of culture in nursing care and identified their perceived cultures and explained how they understood the concept of culture. They also stated their various beliefs and values as determined by their culture.

#### Subtheme 1.1: Description of culture and its importance

When the participants were asked if they thought that culture is an important concept in nursing, that they need to be asked about by the nurses, they agreed as shown in the following responses:

‘Yes it is very, very important.’ (P7, Indian, Muslim)

‘Yes, yes definitely.’ (P21, Indian, Christian)

‘They must ask, … because then you know the person better.’ (P1, Mixed race, Christian)

However, the participants generally seemed to struggle to describe their own cultures. They referred to either home language or racial grouping, religion, and nationality to describe their culture. According to Mokgotlane et al. (2013):

‘Culture is defined as a shared set of norms, values, perceptions, and social conventions that give cohesion to a group, race or community enabling them to live together and function effectively and harmoniously. It is a key influence on how an individual perceives the world and responds to it.’ (p. 12)

In an attempt to show their understanding of their cultures, participants stated the following:

‘Okay, when I …, when I answer that question [*she was asked to identify her culture*], it’s about my language … I speak Tswana.’ (P4, African, Christian)

‘Christian … Probably Indian … we all talk English.’ (P3, Indian, Hindu)

‘I would usually say South African, ne … Ya, a so-called coloured [*mixed race*].’ (P1, Mixed race, Christian)

‘I was schooled in English but … most of my family are Afrikaans … South African basically.’ (P9, Caucasian, Christian)

#### Subtheme 1.2: Values and norms

The participants also struggled to articulate their values and norms, and the needs that emanate from these. The participants’ values and norms were varied showing they were a diverse group. Their values and norms were linked to how they identified their cultures (mainly through religion, race, or ethnicity) and were mainly related to food, dress code, respect, and even kindness. The participants stated the following to show what they valued:

‘Muslims, they don’t eat certain food and if you know that I don’t eat pork, I don’t eat anything that’s cooked out of a Christian’s pot, that’s my culture …’ (P7, Indian, Muslim)

‘We have to cover our whole bodies because a woman’s body is sacred it is not allowed for another man to see.’ (P19, Mixed race, Muslim)

‘The coloured [*mixed race*] culture is just respect.’ (P1, Mixed race, Christian)

‘I just feel that humility is the best way of bringing healing to a person … by being kind and loving and showing and taking a few minutes to chat with a patient.’ (P 21, Indian, Christian)

‘A Christian will teach you that we must love one another and that we must follow the ten commandments.’ (P20, Mixed race, Christian)

The results of a study by Wilson (2010) conducted in the US on nurses and African-American psychiatric patients also showed that both patients and nurses struggled to explain their cultures and lacked specific knowledge about culture. This shows that defining culture is generally a difficult concept to define for everyone, not only for the participants in this study.

### Theme 2: Importance of cultural competence in nursing

Cultural competence was seen as an important concept in nursing by the participants in this study.

#### Subtheme 2.1: Nurses’ cultural knowledge

Most of the patients who participated in this study thought that patients should be asked about their culture by the nurses and that nurses should possess knowledge about their patients’ cultures. One of the patients stated:

‘Yes, I think it’s important for the nurses to know the patient’s culture because if you don’t know my culture, I might think that you treat me unfairly.’ (P15, African, Christian)

Others stated the following regarding the need for nurses to ask patients about their cultures:

‘That is the first thing the nurse must find out about the patient, what is their culture, and if they don’t do that they will just treat you like nothing [*to be disrespected or treated like a worthless person*].’ (P1, Mixed race, Christian)

‘Yes it is very important…they will find out about how the Muslim culture lives [*the Muslim patients’ lifestyle*].’ (P7, Indian, Muslim)

#### Subtheme 2.2: Nurses’ self-awareness

The patients expressed the importance of self-awareness on the part of the nurses, as the patients indicated that mutual respect between nurse and patient could not occur without such awareness. Some of them stated the following when asked if it is important for nurses to have self-awareness and the benefits of this:

‘[*Y*]ou must be self-aware about yourself … Your culture, where do you come from [*where you come from*], what are you, what’s your ancestors about [*what your ancestors are about*]. I think that’s important. If you know what’s important to you, you will apply it to … [*the*] other person.’ (P1, Mixed race, Christian)

‘So they know themselves better and that will help them in understanding other people’s cultures.’ (P9, Caucasian, Christian)

#### Subtheme 2.3: Cultural needs do change

The cultural needs of the participants seemed not to be a priority for some of the participants. This was because of two reasons. Firstly, it was because they have changed because of becoming more modernised or changing their cultural perspective. This is shown by what the following participants stated:

‘We are not to be disrespectful in any way but with modern times things has [*have*] changed. Our dressings are different. Our talking is different because we used to talk the mother language but nobody talks the mother language anymore.’ (P3, Indian, Hindu)

‘I am actually in the middle between black culture and white culture because I am married to a White culture. So yes, if I … I would like to be addressed in a professional way where it will be accommodating, I will say, both. Not black and not white, but general professional, I will prefer that.’ (P16, African, Christian)

Secondly, these patients seemed not to believe they had a right to have their own cultures acknowledged once they were in the hospital, as shown by the participants who stated the following:

‘[*T*]hey [*patients*] have got to leave their culture at home.’ (P3, Indian, Hindu)

‘To be honest it’s not necessary. I feel that people need to be treated the same. You need to have a culture that you treat people with … I mean every company, [*and*] every hospital has their own rules, apply the same rule to everybody. Doesn’t have to be … because we can’t speak Zulu and the other one you speak Chinese, it’s impossible.’ (P16, African, Christian)

### Theme 3: Meeting patients’ cultural needs

Several points were raised by the participants in this study about meeting their cultural needs.

#### Subtheme 3.1: Cultural skill

The participants expressed the need for nurses to show cultural skill. When asked what they thought nurses should ask them about their culture, they stated the following:

‘Like what type of food do you eat? … because different cultures eat different food.’ (P14, African, Christian)

‘[*T*]hey can ask me like, are you a churchgoer or are you belonging to [*believing in*] traditional healers … If she knows I am a Christian she will know I don’t do things that traditional healers do.’ (P15, African, Christian)

‘[*S*]he has to ask me [*about*] my culture and my religion, because when I am very bad [*very ill or my condition deteriorates*] maybe she can call my priest. That’s the first main thing. The priest or your pastor can come in and anoint you if you are very ill. That’s the first thing and then they can call them and say please this patient is very ill, will you please come and see to her ….’ (P20, Mixed race, Christian)

‘They need to ask are you kosher? Kosher means halaal products, number two there has to be prayer facilities, toilet, and water facilities … they have to be always clean … hygiene practices … clean pyjamas and bedsheets.’ (P7, Indian, Muslim)

Sometimes questions asked by nurses during history taking can be superficial or non-specific, and they can miss important information about a patient’s cultural practices that have affected the patient’s condition. One participant stated the following in this regard:

‘I think the first thing the nurse can ask the patient what is the problem and then the patient can tell the nurse what is the problem and then to find out maybe it is something about the culture or not [*nurses need to find out if the patient’s presenting complaint is related to their culture or not*] … Maybe they can ask the patient what you are [*were you*] doing before you come [*came*] here in the hospital? Is there anything that you did it wrong (did wrong) or maybe you take [*took*] it that you think maybe make [*made*] you sick to come here [*nurses need to ask specific questions about what patients took or did before getting ill in order to identify if patients took any cultural medication*].’ (P17, African, Christian)

An important component of cultural skill is that of addressing the patient in a language they are comfortable with, or asking them which language they prefer to be addressed in. This is what one of the participants had to say in this regard:

‘[*W*]hat can I say, we get spoken to in a common language so they don’t really ask if, what or know the type of religion [*patient’s background*] or culture.’ (P14, African, Christian)

#### Subtheme 3.2: Cultural encounters

The study participants raised the need to have cultural encounters with nurses so that they could learn more about their cultures. This was shown in the following comments:

‘[*T*]he nurse needs to communicate with the patient, talk to the patient, be friendly and know the background and then the nurse will be able to explain them because if they are doing the wrong things the nurse will be able to explain [*to*] them no this is not right. You need to do this. You need to take your medication at a certain time and okay fine if you’re doing some herbal thing it’s fine but some of them are doing things that are very illegal [*very illegal things*].’ (P21, Indian, Christian)

‘It’s to communicate to the patient or person, communication is playing [*plays*] a good role in understanding each other.’ (P1, Mixed race, Christian)

‘I think to learn he must ask the patient what is his culture and then the patient will tell them and then he can understand what is the culture of the patient.’ (P17, African, Christian)

Participants also thought the nurses did not have time to have cultural encounters with them and suggested other ways nurses could learn about their patients’ cultures. This is illustrated by the participants who stated the following:

‘[*I*] don’t think there’s time for them to kind of know about our cultures ….’ (P14, African, Christian)

‘Reading … watching the different postures how Muslim patients pray and respecting … observing.’ (P7, Indian, Muslim)

‘Watching TV and learning more from TV also.’ (P21, Indian, Christian)

#### Subtheme 3.3: Cultural practices or beliefs

The cultural needs of participants in this study were influenced more by religion than their indigenous cultures as most were Christian and thought that some of their indigenous traditional beliefs and practices could be harmful and detrimental to one’s health, hence they could not be practised in a hospital setting, as some participants stated:

‘Anything to do with medication, like when you are pregnant you have to drink Isihlambezo [*a herbal tonic used by Zulu pregnant women*]. I mean that can affect the baby depending on the medication I wouldn’t agree with stuff [*things*] like that.’ (P16, African, Christian)

‘[*A*]nd that is a bit of a tricky one because every culture has got their belief … So now we, when we come to the hospital, we sort of leave that out for a while…because there is (are) certain days that we fast. We do not have any flesh [*meat*] or egg or anything … and when you come here like today is Tuesday … I will carry it because I will eat but if I have to sleep here tonight and if they have to give me fish tonight to eat I will have said to myself, God please forgive me but this a meal that has been prepared … Yes, they [*patients*] have got to leave their culture at home.’ (P3, Indian, Hindu)

One of the Christian participants stated the following to show her religious beliefs:

‘[*T*]here is sometimes [*there are times*] when I feel, when if I’m not feeling okay [*when I don’t feel well or when feeling sad*], I just take my Bible, when I open my bible there are some verses, [*and*] sometimes I don’t know what is going on, but sometimes I believe that, like if I’m hurting or something, when I just open the Bible and read it, I feel like God is answering me.’ (P4, African, Christian)

However, some of these patients did acknowledge that other patients would need to practise their indigenous traditional practices and that they should be allowed to do so, as long as the health of other patients is not put at risk. This is what some of the participants stated about nurses meeting the needs of such patients:

‘Yes, they, they know it’s impossible but they might need the medication from the traditional healer.’ (P15, African, Christian)

‘Respect their culture, assist them in the way of their culture.’ (P7, Indian, Muslim)

‘[*B*]ut something like physically that doesn’t affect anything, it doesn’t disagree with what they give you [*if traditional medicines don’t negatively interact with medicines given in hospital*], I think those kind [*kinds*] of practices can be allowed. As long as it doesn’t affect anybody else and they are just personal.’ (P16, African, Christian)

Some of the participants even raised concerns about the potential for their needs to be neglected as illustrated by one of the participants who stated the following:

‘[*I*]n the hospital maybe you make a what? A menu for Monday up to Sunday I don’t know like all the food you put [*it*] there. Like today we ate vegetables and beans … Tomorrow we eat what … ngulube [*pork*] and what … vegetable. And the next time so some of them which means you destroy some people their culture [*some people’s culture*].’ (P10, African, Christian)

‘But ja [*yes*], it’s [*they are*] supposed to be free to eat what they want because it’s their … according in their culture too. You supposed to.’ (P9, Caucasian, Christian)

The Muslim patients raised the fact that it is critical to practise cleanliness in their religion and to have prayer facilities. Some participants stated:

‘[*M*]uslim culture is free, clean and the nurses have to learn about the way, to adapt when they are coming to a Muslim patient, considering their lifestyle of dressing, of food, health and cleanliness and types of prayer, which times of the prayer, clothing, also cleanliness and food, very important, that koshered food, and expiry dates on food is also important for Muslims because sometimes the fat can, you know, also cause a problem in there … the prayer facilities that also is very important ….’ (P7, Indian, Muslim)

‘[*E*]ven if the guys go to urinate and like you are on the bed and if I use a bed pan, they must make sure that my clothing is pulled far down, that the urine doesn’t affect me on my clothing, or if it does take effect, I have to change it, because *due to* prayers of five times a day, … so all the time you have to be pure, clean.’ (P7, Indian, Muslim)

Some of the needs raised by patients were in line with what is already practised in hospitals. For instance, the practice of handwashing, carried out in hospitals is supported by Islam (Muslim religion). The World Health Organization (WHO) in its (2009) hand-washing guideline recognises the critical importance of hand-washing in Islam, Judaism, and Sikhism. The guideline also states that Christians and Buddhists also recognise the importance of handwashing.

#### Subtheme 3.4: Role of the nurses and unit managers

The participants explained that when nurses meet patients’ needs on their own without being forced to do so by their managers (possess cultural desire, as explained in Canmpinha-Bacote’s 2002 model), and nurse managers ensure that nurses are culturally competent, their cultural needs are more likely to be met and this could assist in their recovery and improve their satisfaction with the nursing care they receive. This is what they stated about the role of the nurses:

‘[*I*] think maybe, maybe the sister when they get in the ward, they must know there is [*are*] different people in this ward and they don’t have one culture of Christian [*Christianity*] and they must give us the… all of them ask … they must give us the culture that you need [*accommodate all cultures*].’ (P1, Mixed race, Christian)

‘[*I*] need, the nurse must respect my culture and the thing that I want to, maybe when I come to the hospital and I know that maybe I’m going to stay for two days … I must carry my bible and in the morning or just like a … in the night I can read my bible and maybe I must be free of [*with*] the people of [*from*] church when they visit me, I must be comfortable.’ (P20, Mixed race, Christian)

To distinguish the role of the nurse as compared to that of the nurse managers in meeting the patient’s cultural needs, some of the patients stated the following:

‘Actually, it is nurses themselves that deal with the patient, because management is there, the nurse in charge is there, she’s telling them what to do, she’s giving orders, and they [*are*] working on the ground with the …, in the ward with the people.’ (P1, Mixed race, Christian)

‘They have to first … they need to educate their staff, they must educate, have courses so they can understand different cultures, I think it starts from there first. If the staff have knowledge and everything and they put it as a procedure so they can apply it in that way as a standard procedure. But if it is not a procedure it won’t be applied.’ (P16, African, Christian)

Others stated the following about the role of the unit manager:

‘[*T*]he thing is the manager have [*has*] … is … the thing that he or she must do, he must always come to the ward and check what is going on there, yes.’ (P4, African, Christian)

‘So the role of the sister in charge in the ward, she might see if the nurses are treating the patient okay, alright, and then she must also take care of if the patient complaints, she must do her best to help.’ (P15, African, Christian)

Castro and Ruiz (2009) recommend that employers seeking to meet the healthcare demands of a growing diverse population must look at extrinsic values such as cultural competence training, nursing education and certification, and the ability to speak indigenous languages. It is therefore imperative for nursing managers to prioritise cultural competence in nursing units.

#### Subtheme 3.5: Benefits for patients and nurses

The participants also discussed the benefits of meeting patients’ cultural needs for both the patient and the nurse. According to the participants, the patients will benefit when their cultural needs are met by the nurses and nurses will derive some benefit from meeting patients’ cultural needs. The benefits for the patients would be satisfaction with the care they receive which would contribute positively towards their health or wellbeing. This is what some participants answered when asked whether it was beneficial for patients when their cultural needs were met:

‘Yes definitely, because they will feel at home.’ (P21, Indian, Christian)

‘A happy patient recovers quicker.’ (P7, Indian, Muslim)

One of the participants stated the following when asked how the patients would benefit when their cultural needs were met:

‘Spiritually, emotionally, and in their life, because at the end of the day is it something that needs to be done and it affects them otherwise. So, it benefits them in every area.’ (P16, African, Christian)

The participants explained that when a nurse respects patients’ cultures, they in turn are respected by patients. They also thought that nurses would gain more knowledge about the patients’ cultures in the process of meeting these needs. This is what the participants had to say when asked how nurses would benefit from meeting patients’ cultures:

‘[*W*]hat I know of, it will make things even better, not only just for the patient but obviously the nurse will get to know more about the patient as well. That’s how the nurse starts learning different cultures in fact.’ (P7, Indian, Muslim)

‘She is going to respect that nurse.’ (P3, Indian, Hindu)

### Theme 4: Evaluation of nurses’ cultural competence

This theme emerged from the data without a specific question or probes. It revealed the participants’ satisfaction and dissatisfaction with the nurses’ cultural competence.

#### Subtheme 4.1: Some satisfaction for Christians

Most of the Christian participants were satisfied with how their cultural needs were met as shown by some of the participants who stated the following:

‘When they see me read my bible, they don’t have a problem with that.’ (P11, African, Christian)

‘We also had some people from a church who came to pray for us.’ (P4, African, Christian)

‘[*I*] love them [*the nurses*], in the morning when they come in, the nurses, and I think I used to take part with them, when they pray and they sing I love it and even before they begin, it is very nice. Even if they sing, I know because we also sing. I can’t speak the language, but the hymn ….’ (P20, Mixed race, Christian)

This shows that the nurses were able to meet the needs of Christian patients (showed cultural desire) because they shared a religion with the patients.

#### Subtheme 4.2: Dissatisfaction with nurses’ cultural competence

Some of the participants, however, expressed dissatisfaction with the cultural competence of some of the nurses they interacted with during their admission to the hospital or the admission of one of their relatives to the hospital. They verbalised the fact that they were unhappy with the interaction or cultural encounters experienced with nurses because they were either discriminated against (no cultural desire was shown on the part of the nurses) because their culture was different from that of the nurses or the nurses did not cater for any of their cultural needs (they showed no cultural skill) or asked them about their cultures to increase their cultural awareness or cultural knowledge. These participants stated the following:

‘[*B*]ecause he’s light of [*in*] complexion, they didn’t even had [*have*] a …, they treated him so bad, I had such a bad experience with that [*those*] nurses.’ (P1, Mixed race, Christian)

‘Well, because I mean, when you come for … when you do your nursing course, right? They teach you all these things, but what happens? They don’t practice it. Once they’ve got the certificate as a nursing … [*nurse*] to practice what they have been taught, they don’t practice it anymore.’ (P7, Mixed race, Muslim)

‘Well, I think there is basically a lack of communication between [*I*] won’t say between both the patients and nurses because it must come more from the nurses because it is the duty of the nurses to be able to ask the patient which culture they come from, in order to know how to take care of you … they look at the colour of your skin and so they assume…’ (P6, Mixed race, Muslim)

When one of the patients was asked whether he cared about his cultural needs being met by the nurses, he stated the following:

‘Well I do care but, it, it’s like the, they just come in and out you know so I don’t think there’s time for them to kind of know about our cultures and whose, it’s like, what can I say, we get spoken to in a common language, so they don’t really ask If … or know the type of religion or culture.’ (P14, African, Christian)

One of the participants felt some of the cultural practices that were currently being allowed by the nurses could be perceived negatively by some patients. She stated the following:

‘Praying and singing and their dancing and they clapping hands and whatever. Now maybe if the Sister will allow it. But other patients not of that culture [*patients of other cultures*] will not like it.’ (P3, Indian, Hindu)

This shows that patients of other religions (who are not Christian) may feel their cultural needs are not given the same status as the majority of nurses in Gauteng public sector hospitals practise Christianity while on duty in their respective nursing units. Castro and Ruiz (2009) state that patient satisfaction with care is associated with increased compliance with treatment and hence continuity of care. This means that patients who feel that their cultural needs are met in a hospital are more likely to comply with the treatment given in the hospital, leading to better patient outcomes. It is therefore very important to ensure the satisfaction of all patients regardless of which cultural or religious group they belong to.

## Discussion

Patient perceptions about cultural competence were explored and described in Gauteng, a cosmopolitan province where cultural diversity is prevalent in nursing units of public sector hospitals which often poses difficulties for nurses and patients. The findings showed that patients in public sector hospitals in Johannesburg, Gauteng, think that culture and cultural competence are important concepts in nursing. When soliciting patient perceptions about the cultural competence of nurses, the constructs of the chosen model seemed comprehensive and the themes solicited could be related to these constructs. The first theme related to the understanding of one’s own culture indicated that even though the participants struggled to define their own cultures, their definition of the concept was similar to that stated in Mokgotlane et al. (2013). The importance of culture and nurses becoming culturally competent is well-established in literature as supported by Alizadeh and Chavan (2015).

This importance was also echoed by the participants of this study in the second theme (Patients’ understanding of culture). However, this theme also highlighted the fact that patients’ cultural needs do change over time and that in some places (like hospitals) patients may not need to have their cultural needs met. Nevertheless, the provision of culturally competent care, according to Castro and Ruiz (2009), is important as it leads to negotiation, mutual exchange of information with patients, and improved patient-provider communication. Still, under this theme, the participants stated that nurses need to have self-awareness (cultural awareness) because if they know about their own cultures, they will also be able to understand their patients’ cultures.

The participants identified their cultural needs which should be met by nurses in the third theme when they are interacting or communicating with patients during their hospital admission. Dwamena et al. (2012) state that communication difficulties in healthcare can occur when health professionals choose to focus on diseases and the management thereof rather than on people, their lives, their background, and their context. Nurses must therefore focus on their interaction with patients to understand their cultural background and cultural needs. The nurses’ cultural awareness (self-assessment and reflection on their background), cultural knowledge (the knowledge they possess about patients’ cultures), and cultural skill (their ability to collect relevant cultural data from patients) are components of cultural competence. If the communication between nurses and patients lacks cultural competence, it can affect patient outcomes and consequently, the absence of cultural competence in nursing units could have a dire impact on patients. This is because communication is critical in ensuring patients can express their needs and receive the life-saving assistance they require from nurses.

In a study by Castro and Ruiz (2009) conducted in the US, patients were mostly satisfied by the cultural competence of nurses who were of their race, had received cultural competence training, had a master’s degree, and could speak their patients’ language. The opposite could also be true. The fourth theme showed that Christian patients in this study were most satisfied with how their cultural needs were met; however, patients from other religions felt discriminated against because most of the nurses practise Christianity with some aspects such as morning prayers carried out in the workplace in public sector hospitals in Gauteng. These patients were not satisfied with the nurses’ cultural competence. The unit manager’s role in this regard is to ensure that patients receive culturally sensitive care, as shown in the third theme. However, the nurses also need to exhibit cultural desire as per the Campinha-Bacote model (2002). The findings of this study indicated that nurses showed cultural desire (having morning prayers) but only for the patients who practised Christianity. Non-Christian patients voiced their dissatisfaction with nurses who do not spend time with them to understand their needs. A study on the cultural competence of midwives in South Africa indicated that they are willing to learn about the cultural needs of their patients, thus showing cultural desire. These participants voiced their concern that nurses are not adequately trained which supports the recommendation of this study that nursing units should improve the cultural competence of nurses (Shopo et al. 2023). According to Armstrong et al. (2010), managing cultural diversity in an organisation leads to a successful organisation, where the staff members are productive and the objectives of the organisation are met.

The findings of the study show that nurses do not always cater to patients’ cultural needs especially when the patients’ religions differ from their own. The participants indicated that they would have a better experience and be more satisfied if nurses were culturally competent at all levels indicated in the Process of Cultural Competence in the Delivery of Health Care Services model by Campinha-Bacote (2002). Further research in other provinces of South Africa to explore the same patient perceptions of cultural competence as different provinces of South Africa have varying levels of multiculturalism is necessary. Nurses need to familiarise themselves with the various cultures of patients and strive to meet the cultural needs of all patients. The Gauteng Department of Health should invest in cultural competence training for nurses in public sector hospitals to increase the nurses’ awareness about cultural competence and to assist nurses with implementing measures to improve their cultural competence. Nursing students in both undergraduate and post-graduate levels in Gauteng should be educated about cultural competence.

An interesting finding in the study was that most patients did not mention any needs related to the indigenous cultures. This was because most of the study participants were Christians and their needs were mostly influenced by religion rather than indigenous cultures. This highlights the importance of religious and spiritual practices as part of a patient’s cultural needs when admitted to a hospital, as supported by literature (Ramírez Stege & Godinez 2022; Roh, Burnette & Lee 2018). Shopo et al.’s (2023) study on midwives’ experiences of providing culturally competent care revealed that patients are secretive about their cultural practices during labour. This could have also applied to some of the participants of this study who were not comfortable disclosing their cultural practices.

The limitation of this study was that it was conducted in only one region of one province in South Africa, which limits the generalisation of the findings to other parts of the country. Most of the participants were outpatients and even though they all had the experience of a hospital admission in the past, they could have forgotten about some aspects of their hospital experience.

## Conclusion

The main finding of this study conducted in three public sector hospitals in Gauteng, was that patients think that culture and cultural competence are important concepts and expect their cultural needs to be met when they are admitted to the hospital or interacting with nurses. This means, to offer good quality nursing care to patients, nurses cannot ignore patients’ cultural needs; instead they need to seek to become culturally competent. Participants in the study indicated that some of these needs were not met by nurses and thought that nurses should become culturally self-aware. Cultural desire was observed for Christian patients and thus only partially met. The main concern raised was that of cultural practices around religion and the need for nurses to understand and have the skill to accommodate the cultural needs of their patients such as food, language preferences, and religious practices. The finding of this study showed that nurses need to embark on a process of becoming culturally competent to meet these needs as participants felt this would improve the quality of care and the satisfaction patients have with the care they receive.
